# The Genomic Basis of Intrinsic and Acquired Antibiotic Resistance in the Genus *Serratia*

**DOI:** 10.3389/fmicb.2018.00828

**Published:** 2018-05-11

**Authors:** Luisa Sandner-Miranda, Pablo Vinuesa, Alejandro Cravioto, Rosario Morales-Espinosa

**Affiliations:** ^1^Departamento de Microbiología y Parasitología, Facultad de Medicina, Universidad Nacional Autónoma de México, Mexico City, Mexico; ^2^Centro de Ciencias Genómicas, Universidad Nacional Autónoma de México, Cuernavaca, Mexico

**Keywords:** resistome, genus *Serratia*, antibiotics resistance genes, intrinsic resistance, acquired resistance

## Abstract

*Serratia marcescens*, a member of the *Enterobacteriaceae* family, was long thought to be a non-pathogenic bacterium prevalent in environmental habitats. Together with other members of this genus, it has emerged in recent years as an opportunistic nosocomial pathogen causing various types of infections. One important feature of pathogens belonging to this genus is their intrinsic and acquired resistance to a variety of antibiotic families, including β-lactam, aminoglycosides, quinolones and polypeptide antibiotics. The aim of this study was to elucidate which genes participate in the intrinsic and acquired antibiotic resistance of this genus in order to determine the *Serratia* genus resistome. We performed phylogenomic and comparative genomic analyses using 32 *Serratia* spp. genomes deposited in the NCBI GenBank from strains isolated from different ecological niches and different lifestyles. *S. marcescens* strain SmUNAM836, which was previously isolated from a Mexican adult with obstructive pulmonary disease, was included in this study. The results show that most of the antibiotic resistance genes (ARGs) were found on the chromosome, and to a lesser degree, on plasmids and transposons acquired through horizontal gene transfer. Four strains contained the *gyrA* point mutation in codon Ser83 that confers quinolone resistance. Pathogenic and environmental isolates presented a high number of ARGs, especially genes associated with efflux systems. Pathogenic strains, specifically nosocomial strains, presented more acquired resistance genes than environmental isolates. We may conclude that the environment provides a natural reservoir for antibiotic resistance, which has been underestimated in the medical field.

## Introduction

Human infections caused by members of the genus *Serratia*, most commonly by *Serratia marcescens*, were not well identified until the latter half of the 20th Century, probably due to the taxonomic difficulty in describing the species. *S. marcescens* and to a lesser extent, other members of this genus, are now considered opportunistic nosocomial pathogens causing an array of infections including meningitis, sepsis, urinary tract infections, skin infections, bloodstream and respiratory infections, as well as being important ocular pathogens (Engelhart et al., 2003; Shanks et al., 2013; Wu et al., 2013; Gupta et al., 2014). *Serratia* species harbor several virulence factors including hemolysins (ShlAB), Quorum-Sensing proteins (LuxI-R), biofilm development proteins (BsmB) (only seen in *S. marcescens*), phospholipases (PhlA), peptidases (Clp), metalloproteases, chitinases (ChiABC), siderophores and hemophores (HasA), the lipopolysaccharide LPS, and motility and adherence factors such as flagella and fimbriae (Mahlen, 2011). *S. marcescens* is among the 10 most recovered pathogens in hospitals worldwide (Mahlen, 2011; Bertrand and Dowzicky, 2012) and has been cultured from a variety of sources including disinfectants, pressure transducers, bronchoscopes, multi-dose medication vials, contaminated antiseptic solutions, fentanyl-containing fluids, contaminated MgSO_4_ and contaminated saline syringes among others (Sunenshine et al., 2007; Liu et al., 2011; Chiang et al., 2013; Merkier et al., 2013; Šiširak and Hukić, 2013; Liou et al., 2014; Hervé et al., 2015; Dawczynski et al., 2016; Morillo et al., 2016; Vetter et al., 2016). Historically, outbreaks of *S. marcescens* have been reported since 1950 and have been considered nosocomial in origin (Mahlen, 2011). Recent *S. marcescens* outbreaks have been reported mostly in North America and Europe, probably due to more efficient surveillance systems in those regions. The majority of the outbreaks occurred in neonatal ICUs, cardiac surgical ICUs, orthopedic clinics and dialysis units. There have been some *S. liquefaciens* outbreak reports in past years in various countries (Grohskopf et al., 2001). Pathogenic strains from other *Serratia* species have been isolated from individual patients were not associated with any epidemic outbreak, as seen in the case of a *S. rubideae* isolated from the sputum of a patient in China (Yao et al., 2016). There is a large amount of published data about *Serratia* epidemiology and resistance patterns among human populations worldwide. The *S. marcescens* strains recovered in UCIs from 6 different geographic regions (Africa, Asia, the Asia-Pacific Rim, Europe, Latin America and North America) show a similar resistance/susceptibility pattern in all regions: resistance to all penicillins and susceptibility to all carbapenems. However, Latin American strains show a higher percentage of resistance to all antibiotic tested (Bertrand and Dowzicky, 2012).

Many members of the *Serratia* genus contain genes related to antimicrobial resistance, which confer resistance to β-lactam, aminoglycosides, quinolones, macrolides and polypeptide antimicrobials. Intrinsic resistance in microorganisms is conferred by antibiotic resistance genes (ARGs), including genes associated with efflux pumps, which are present on the chromosome and shared by members of the same species or genus. Acquired resistance is conferred by the gains of novel resistance genes via horizontal gene transfer (HGT) or by mutations of particular chromosomal genes. Generally, horizontally transferred resistance genes are located on mobile genetic elements, such as plasmids, integrons, transposons or genomic islands (Blair et al., 2014; Hu et al., 2015), and can be defined as any segment of DNA that can translocate from one part of the genome to another or, between genomes (Van Hoek et al., 2011).

The aim of this study was to identify the genes responsible for intrinsic and acquired multidrug-resistance (resistome) of the genus *Serratia* using 32 *Serratia* spp. genomes deposited in the NCBI database belonging to strains isolated from different ecological niches from 19 countries and 4 continents. We also included the *S. marcescens* SmUNAM836 strain, which was previously sequenced by our group.

## Materials and Methods

### Bacterial Strains and Genomes

Comparative genomic analysis was carried out using 32 whole, sequenced *Serratia* genomes retrieved from NCBI’s GenBank and RefSeq repositories (**Supplementary Table S1**), including the *S. marcescens* SmUNAM836 strain, which was sequenced at the Yale Center for Genome Analysis (YCGA) and assembled and annotated by our group (Sandner-Miranda et al., 2016).

### Computing Conservative Consensus Core- and Pan-Genomes

High stringency homologous gene clusters were computed with the GET_HOMOLOGUES (Contreras-Moreira and Vinuesa, 2013) software package by imposing a minimum of 90% query coverage on the all-against-all BLASTP results and performing a PFAM-domain scanning on each sequence to ensure that all homologous gene clusters contain the same domain composition and order. Clustering was performed with the BDBH (-e -D), COGtriangles (-G -t 0 -D) and OMCL (-M -t 0 -D) algorithms implemented in GET_HOMOLOGUES (Contreras-Moreira and Vinuesa, 2013) and the indicated parameters. A consensus core-genome was computed with the aid of the compare_clusters.pl script (-t 34) from the clusters generated by each of the tree algorithms, as detailed elsewhere (Vinuesa and Contreras-Moreira, 2015). Similarly, a consensus pan-genome was computed from the COGtriangles and OMCL clusters using the ‘-m -t 0’ parameters, to generate the pan-genome matrix reporting clusters of all sizes.

### Estimating a Robust Maximum-Likelihood Core-Genome Phylogeny for the Genus *Serratia*

A core-genome phylogeny was estimated under the maximum-likelihood (ML) optimality criterion using the consensus core-genome clusters computed by GET_HOMOLOGUES (Contreras-Moreira and Vinuesa, 2013) as described in the previous section and passing them to the GET_PHYLOMARKERS (Vinuesa et al., 2018, in revision) software suite, which was run in default mode (-R 1 -t DNA). The latter is freely available on GitHub^1^. Briefly, the GET_PHYLOMARKERS pipeline was used to select core-genome loci with optimal phylogenetic attributes, namely those passing the phi recombination test (Bruen et al., 2006), producing tree topologies and branch-lengths not significantly deviating (kdetrees test) from the expected distribution of these parameters under the multispecies coalescent (Weyenberg et al., 2014) and displaying average branch support values >0.7 (see Vinuesa et al., 2018 for the details). The clustal–omega (Sievers et al., 2011) codon alignments passing these filters were concatenated and a ML phylogeny estimated with IQ-TREE 1.6.1 (Nguyen et al., 2015) using the best fitting model and selecting the phylogeny with the highest likelihood score from those found among independent searches.

### Computing Pairwise Core-Genome Average Nucleotide Identity Values From OMCL Clusters (cgANIb-OMCL)

Pairwise cgANIb values were computed from the BLASTN alignments identified by OMCL as belonging to the core-genome with the aid of the get_homologues.pl script, run with the ‘-A -a CDS’ parameters. The resulting cgANIb-OMCL matrix was then displayed as an ordered, bi-dimensional heatmap with the aid of the plot_matrix_heatmap.sh script, distributed with the GET_HOMOLOGUES package.

### Phylogeny of *Serratia* spp. Based on Its Pan-Genome

A parsimony pan-genome phylogeny for the 32 *Serratia* genomes was estimated from the consensus pan-genome matrix of presence/absence data for homologous clusters created with the aid of the compare_clusters.pl script from the GET_HOMOLOGUES (Contreras-Moreira and Vinuesa, 2013) package, run with the -m -t 0 -T parameters, which calls PARS from the PHYLIP suite. The total number of resistance determinants and the number of acquired and intrinsic resistance genes, including the number of efflux pump genes identified in each genome, are indicated on the tree.

### Identification of Antimicrobial Resistance Genes in the *Serratia* Genus

The whole genome for all strains was analyzed using BLASTN to identify ARGs (Camacho et al., 2009). Searches were also made against locally maintained versions of the CARD (a rigorously curated collection of known resistance determinants) (McArthur et al., 2013) and ResFinder (Zankari et al., 2012) databases. Mutations of genes associated with resistance were searched with BioEdit 7.2.5 (Hall, 1999) and MEGA7 softwares (Kumar et al., 2008). We aligned the 32 *Serratia* whole genomes using Mauve 2.4.0 (Darling et al., 2010) in order to enable identification of the chromosomal genes associated with intrinsic antibiotic resistance by sequence homology. Mutations of genes *gyrA* and *gyrB* (resistance to quinolones), *murA* (resistance to fosfomycin) and *folP* (resistance to sulfamethoxazole) were assessed for acquired resistance using BioEdit 7.2.5. In addition, each genome was screened for additional and strain-exclusive ARGs. In order to identify acquired genes on plasmids or integrons, we used BLASTN (Altschul et al., 1990), Mauve 2.4.0. and ISfinder (Siguier et al., 2006).

### Relationship Between the Isolation Source of Each *Serratia* Species and Its Resistance Genes Content

We performed a Principal Coordinate Analysis (PCoA) (Gower, 1998) in order to visualize similarities and dissimilarities among the 32 *Serratia* spp. resistomes and their relationship with their ecology. We grouped the isolation sources of the strains into three main categories: pathogenic, environmental and symbiotic strains. The environmental strains were divided into three sub-groups: environmental strains associated with soil and plants, environmental strains isolated from water and environmental strains isolated from food sources. Analysis was performed for each *Serratia* strain based on antibiotic resistance gene content.

## Results

### A Robust and Highly Resolved Core-Genome Phylogeny for the Genus *Serratia*

**FIGURE 1:**
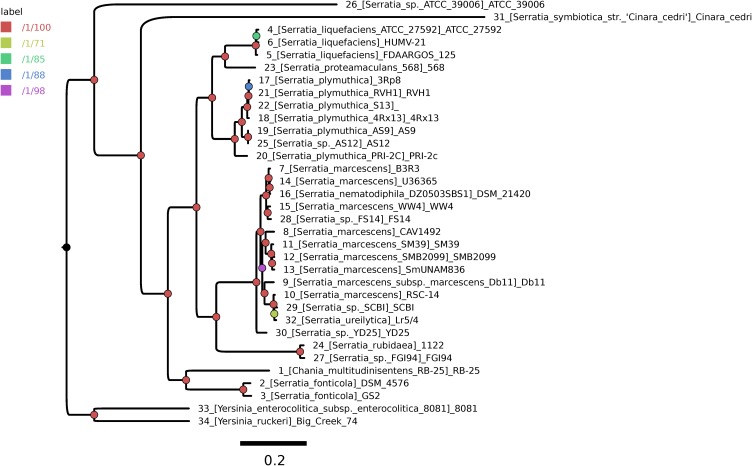
Core-genome phylogeny estimated using the GET_PHYLOMARKERS pipeline to compute a maximum-likelihood (ML) tree from the concatenated supermatrix of 264 top-scoring codon alignments.

A highly stringent consensus core-genome of 396 genes was computed for the 32 *Serratia* spp. genomes and two *Yersinia* spp. genomes, used as outgroup sequences (**Supplementary Figure S1**). We used the GET_PHYLOMARKERS pipeline to compute a ML core-genome phylogeny from the concatenated supermatrix of 264 top-scoring codon alignments passing the sequential filters imposed by the pipeline. **Figure 1** shows the ML tree estimated under the best-fit GTR+F+ASC+R4 model (ln*L* score: -1793389.569) and rooted at the branch subtending the clade grouping the two *Yersinia* genomes. The tree is highly resolved, as indicated by the approximate Bayesian and UFBootstrap values computed for each bipartition by IQ-TREE. The two most basal branches of the ingroup clade correspond to environmental (ATCC39006) and endosymbiotic (*S. symbiotica*) organisms. The latter has a strongly reduced genome, containing only 672 CDSs, being also atypical due to its low G+C content, which is only ∼ 29%. Seven lineages were resolved further inside the tree, which are consistent with the species-level classification of the genome sequences (numbered branches in **Figure 1**). However, several taxonomic inconsistencies were identified in the *S. marcescens* clade, which tightly groups *S. nematodiphila* DSM 21420 and *S. ureilytica* Lr5/4 nested within it. In addition, the species-tree presented in **Figure 1** strongly supports the classification of strain AS12 as a member of *S. plymuthica*, strains FS14, SCBI and YD25 as members of *S. marcescens* and strain *Serratia* sp. FGI94 as *S. rubidea*. These reclassifications were fully supported by average core-genome identity values (cgANIb) computed from the pairwise BLASTN alignments used by OMCL to cluster the core-genome loci. In all cases these strains had a cgANIb value >98% when compared to the closest named species (**Supplementary Figure S2**), as indicated in the previous sentence and shown on the ML species-tree depicted in **Figure 1**.

### Phylogenetic Structure of *Serratia* spp. Pan-Genome and the ARGs Number

The parsimony pan-genome tree was derived from a consensus presence/absence matrix of homologous genes based on the clustering algorithms OMCL and COGtriangles. The Venn diagram shows the number of gene clusters of the 32 *Serratia* spp. pan-genome (12,347 clusters) (**Supplementary Figure S3**) that were used to generate the pan-genome matrix and the phylogeny.

**FIGURE 2:**
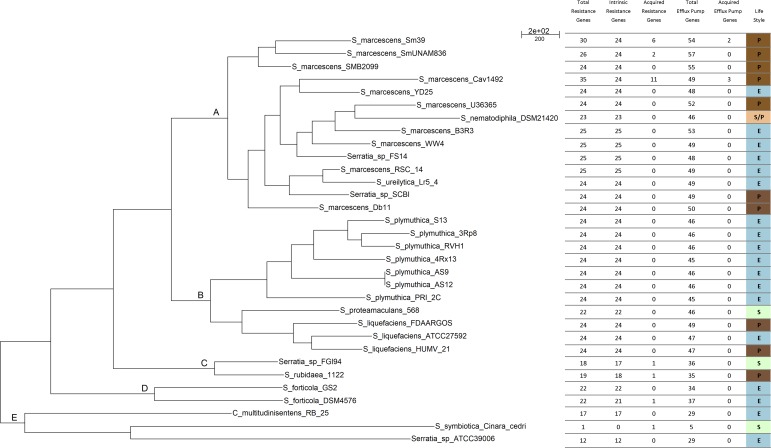
Parsimony pan-genome tree for 32 *Serratia* genomes derived from presence/absence of homolog genes in a consensus pan-genome matrix computed by the COGtriangles y OrthoMCL clustering algorithms. This phylogeny was the most parsimonious tree found in a tree search made with PARS from the PHYLIP suite. The tree has a total length of 20,453 steps. Capital letters A-E indicate the phylogenetic groups.

The pan-genome tree shows five main groups (**Figure 2**). The results of the phylogenetic analysis placed all the *S. marcescens* strains in group A, together with the misclassified *S. ureilytica*, an environmental strain from a geothermal spring and *S. nematodiphila* DSM 21420, a nematode symbiont and an insect pathogen and 3 uncharacterized strains: *Serratia* sp. YD25, *Serratia* sp. FS14 and *Serratia* sp. SCB1. All these strains should be re-classified as *S. marcescens*, based on the evidence gained from the core-genome phylogeny (**Figure 1**) and the cgANIb data (**Supplementary Figure S2**). Most of the acquired ARGs and efflux pump genes are found in strains of this group. Group B consists of two sub-groups: one with the strains belonging to all *S. plymuthica* strains; and the other with the *S. liquefaciens* and *S. proteamaculans* strains, which are all environmental strains, with the exception of *S. liquefaciens* HUMV-21 and *S. liquefaciens* FDAARGOS 125, which are both nosocomial strains. Group C comprises only two strains, *Serratia* sp. FGI94, a fungus symbiont and *S. rubidaea* 1122 isolated from a patient in China. The former has a cgANIb value of 99.2 when compared to the latter, and hence strain FGI94 should be classified as *S. rubidea. S. fonticola* GS2 and *S. fonticola* DSM4576 are placed in group D, both environmental strains. Finally, group E, holds 3 distantly related strains: *Chania multitudinisentens* isolated from the soil of an ex-landfill site; *Serratia* sp. ATCC 39006 isolated from water; and the co-obligate aphid symbiont, *S. symbiotica* ‘Cinara Cedri’. The genomes of these 3 strains harbor the fewest ARGs on their chromosomes. Their cgANIb values are <85% with respect to the other species in the main *Serratia* spp. cluster, and therefore their classification as members of the former genus is questionable.

Phylogenetically, *S. marcescens* SmUNAM836 and *S. marcescens* SM39 are closely related with both genomes sharing the same intrinsic ARGs and efflux pump genes (Iguchi et al., 2014) but different acquired ARGs.

### Identification of Antimicrobial Resistance Genes in the 32 *Serratia* spp. Genomes

Using the CARD database, we looked for ARGs reported to be associated with enterobacteria and more specifically with *Serratia* spp., which were identified on the genomes of the *Serratia* spp. strains using BLASTN. We then aligned the 32 genomes using Mauve 2.4.0 and performed BLASTN to look for the percentage of identity and coverage between the different ARGs. The sequence alignments showed that those genes that appeared to be homologous in Mauve, shared more than 70% of identity between them when performing BLASTN; this, together with the use of global multiple sequence alignments, makes our identification of ARGs more robust (Pearson, 2013). Strain specific ARGs and efflux pump components were detected running a blast search against the CARD database using the following parameters: coverage = 95/90% and identity = 70/50%, respectively.

### Frequency of ARGs According to Each Class of Antibiotic in the *Serratia* spp. Resistome

**FIGURE 3:**
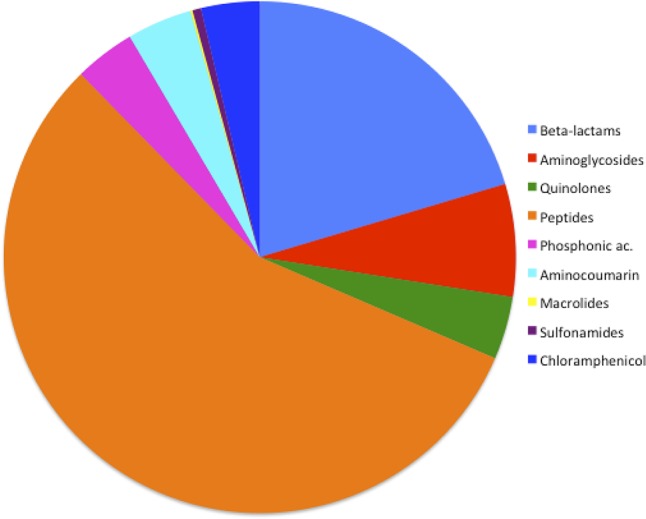
Frequency of ARGs according to the antibiotic class in *Serratia* spp. resistome.

The general frequency of ARGs found in the 32 *Serratia* spp. genomes (efflux pumps not included in this analysis) according to the class of antibiotic was: 0.13% for macrolides; 0.54% for sulfonamides; 3.69% for chloramphenicol; 3.83% for phosphonic antimicrobials; 3.96% for quinolones; 4.1% for aminocoumarin antibiotics (novobiocin); 7.11% associated with aminoglycoside resistance; 20.38% for β-lactam, 56.22% for antimicrobial polypeptides (**Figure 3**). It is not surprising that the highest frequency of ARGs corresponded to the genes that confer resistance to polypeptide antibiotics since *S. marcescens* and the whole genus has a natural resistance to polymyxin B (Olaitan et al., 2014). The second highest frequency in our sample corresponded to β-lactam resistance genes. This fact was expected due to the high number of β-lactamases acquired through HGT in plasmids or transposons found in this genus. The importance of β-lactam resistance genes is reinforced with the fact that up to 1,000 genes related to β-lactam resistance have been described and classified in recent years, among them many new alleles and new genes found in remote geographical niches (Allen et al., 2009; Bush and Jacoby, 2010). In our analysis, the fact that the β-lactamases acquired on plasmids were found exclusively in nosocomial bacteria, except for *bla*_*ACT*-28_, which was identified in an environmental strain associated with plants, is a noteworthy observation (**Supplementary Table S3**). HDT has been shown to play a major role in the transmission and evolution of β-lactam resistance genes in pathogenic and environmental enteric bacteria (Davies and Davies, 2010; Iredell et al., 2016).

### Total Antibiotic Resistance Genes

**FIGURE 4:**
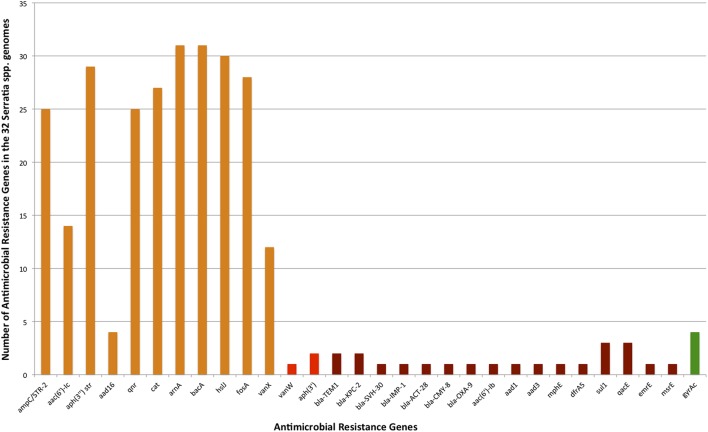
Number of intrinsic and acquired ARGs identified in the 32 *Serratia* genomes (efflux pump genes not included). Intrinsic ARGs in orange color, acquired in red, and acquired by mutation in green.

**FIGURE 5:**
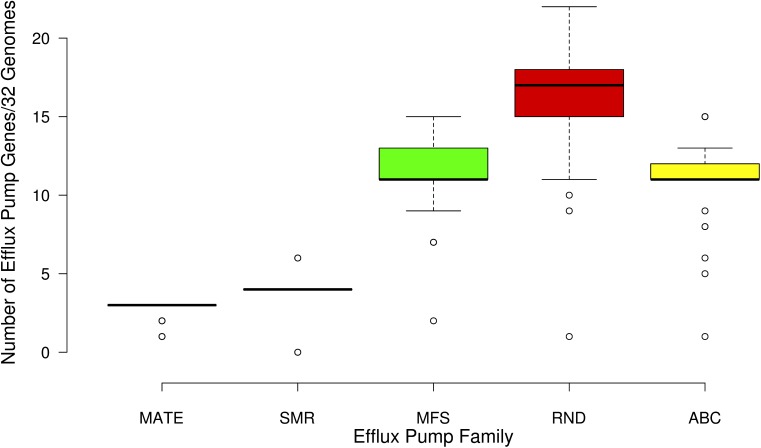
Box plot showing the number of genes associated with the five families of efflux pumps identified in the 32 *Serratia* genomes: multidrug and toxic-compound extrusion family (MATE), the small multidrug resistance family (SMR), the major facilitate superfamily (MFS), the resistance nodulation division family (RND), and the ATP binding cassette superfamily (ABC).

A total of 123 different classes of genes associated with intrinsic and acquired antibiotic resistance were found among the 32 *Serratia* species (detailed information is shown in **Supplementary Table S2**). These ARGs include genes that encode modifying enzymes, antibiotic hydrolysis enzymes, efflux pumps, porines, regulatory proteins, genes with mutations that confer antibiotic resistance, and alleles of some genes and efflux pumps. From the 123 total resistance genes: 33 are intrinsic resistance genes; 16 are acquired resistance genes [13 on plasmids (**Supplementary Table S3**), 2 on transposons, and the gene *gyrA* with the Ser83 mutation that confers quinolone resistance] (**Figure 4**); and 74 are genes associated with efflux systems, from which 3 were localized on plasmids (**Figure 5**).

### Intrinsic Resistance Genes

Intrinsic resistance comprises all of the inherent properties provided by the characteristics of a particular microorganism that limit the action of antimicrobials (Fernández and Hancock, 2012). Most of the genes related to antimicrobial resistance are encoded on the chromosome of the *Serratia* genus. In most genomes, we identified the Ambler class C β-lactamase gene *ampC*, which confers resistance to all penicillins, as well as to 3rd generation cephalosporins and aztreonam when overexpressed (Paterson and Bonomo, 2005), in addition, we found all its regulatory genes: *ampD, ampE, ampG*, and *ampR* (MacDougall, 2011). A class A β-lactamase gene *bla_CTX-M_* and class C β-lactamase gene *bla*_*ACT*-29_ were found only in the two *S. fonticola* genomes. Of the 14 ARGs associated with β-lactam resistance, 10 were found in *S. marcescens* CAV1492, a nosocomial strain (**Supplementary Table S2**).

Three aminoglycoside resistance genes, the aminoglycoside phosphotransferase gene *aph(3″)*, which confers resistance to streptomycin, and the aminoglycoside acetyltransferase gene *aac(6′)-Ic* to amikacin and tobramycin (Shaw et al., 1992) were present on most of the chromosomes studied. We found the aminoglycoside nucleotidyltransferase gene *aadA16* in only 4 genomes from environmental strains. *S. symbiotica*, and *C. multitudinisentens* RB-25 did not present any of these genes.

We identified the *qnr* gene that confers quinolone resistance in the chromosome of 25 strains. Six different alleles of this gene were identified: allele *qnr*B15 in the *S. phymutica* group, allele *qnr*B23 in the *S. marcescens* and *S. nematodiphila* group, *qnr*B31 also in the *S. marcescens* group, *qnr*B32 in the *S. liquefaciens* and S. *proteamaculans* group, *qnr*B37 in the *S. marcescens* and *S. ureilytica* (reclassified as *S. marcescens*) group, and *qnr*B57 in the rest of the *S. phymuthica* strains. Due to the fact that this gene is unique to the *Serratia* spp. chromosome, it could be the possible source of Qnr determinants for the plasmid-encoded Qnr in other species of bacteria (Velasco et al., 2009).

*Serratia* spp. is intrinsically resistant to polymyxin B. Polymyxin B resistance in this genus can be explained by the presence of the *arnBCADTEF* operon, the two-component systems *pmrAB/phoPQ* and the regulatory gene *mgrB* in all strains, except for *S. symbiotica* ‘Cinara Cedri’. Activation of the two-component systems is set off by environmental stimuli that result in an overexpression of LPS-modifying genes (Lin et al., 2014; Olaitan et al., 2014) and leading to polymyxin B and colistin resistance.

Besides the above mentioned antibiotics, the genus *Serratia* is intrinsically resistant to other classes of antibiotics: 31 out of 32 strains have the *bacA* gene on their chromosomes, conferring resistance to bacitracin, another polypeptide antibiotic. In addition, 30 strains harbor the gene *hslJ* and 28 strains the gene *fosA*, which confer novobiocin and fosfomycin resistance, respectively. The chloramphenicol acetyltransferase *cat* gene, which confers resistance to chloramphenicol is also present in 27 strains (Potrykus and Wegrzyn, 2001; Schwarz et al., 2004). Only 12 strains, mostly environmental, possess the vancomycin resistance gene *vanX* on its chromosome (detailed information is shown in **Supplementary Table S2**).

### Intrinsic Resistance Conferred by Efflux Pumps

Active drug extrusion outside of the bacterial cell is one of the most common mechanisms associated with resistance. We identified 74 different genes related to the 5 efflux pump families: the multidrug and toxic-compound extrusion family (MATE), the small multidrug resistance family (SMR), the major facilitator superfamily (MFS), the resistance nodulation division family (RND), and the ATP binding cassette superfamily (ABC) (**Supplementary Table S2**).

Some of these efflux pumps have been described previously in the *Serratia* genus and have homolog in other bacteria: the SsmE efflux pump of the SMR family (conferring resistance to ethidium bromide) is homologous to the *E. coli* EmrE (Minato et al., 2008); SmfY of the MFS family (conferring resistance to norfloxacin, acriflavine and ethidium bromide) homologous to QacA of *Staphylococcus aureus* (Shahcheraghi et al., 2007); SdeXY (conferring resistance to erythromycin, tetracycline, norfloxacin, benzalkonium chloride, ethidium bromide, acriflavine, and rhodamine); SdeAB (conferring resistance to fluoroquinolones) and SdeCDE (conferring resistance to novobiocin) all belonging to the RND family and having a high degree of homology with the AcrAB-TolC, OqxAB and MdtABC efflux pumps of *E. coli* respectively (Chen et al., 2003; Kumar and Worobec, 2005; Begic and Worobec, 2008); and finally, SmdAB (conferring resistance to norfloxacin, tetracycline, and tetraphenylphosphonium chloride) of the ABC superfamily, an homolog of MdlAB of *E. coli* (Matsuo et al., 2008).

Other homologous genes of efflux systems from different bacteria that confer resistance to several kinds of antibiotics and antiseptics were identified on the 32 genomes: 2 alleles of the aminoglycoside efflux pump AcrD from the RND family (Rosenberg et al., 2000); 2 quinolone efflux pumps, DinF and MdtK, from the MATE family (Brown et al., 2007); SugE, a SMR family efflux pump which confers resistance to quaternary ammonium compounds (Chung and Saier, 2002); 9 different MSF family efflux pumps, among them two alleles of EmrAB conferring resistance to thiolactomycin (Furukawa et al., 1993), Bcr to bacitracin (Bernard et al., 2005), Fsr, an homolog of *E. coli* RosA, which confers fosmidomycin resistance, and MdfA, which confers resistance to chloramphenicol (Heng et al., 2015). The efflux pump MacAB from the ABC superfamily, which confers resistance to macrolides (Lu and Zgurskaya, 2012; Fitzpatrick et al., 2017) was also identified on the chromosomes of the *Serratia* spp. genomes, and the efflux pump TetA from the MFS family conferring resistance to tetracycline (Aldema et al., 1996). In addition, 16 genes belonging to 8 efflux systems had alleles on different chromosomal loci, including copies on genomic islands, for example, the aminoglycoside efflux protein AcrD, a transporter belonging to the resistance-nodulation-division family (RND family), which has three different alleles (*acrD, acrD*′, and *acrD*″) distributed among the 32 *Serratia* genomes. The nucleotidic identity percentage between *acrD* and *acrD*′ is 67.2%, while it is 60.9% between *acrD* and *acrD*″ and 62.6% between *acrD*′ and *acrD*″. Two of the nosocomial strains, SmUNAM836 and Sm39, were the only strains that presented all three alleles.

The majority of the genes associated with efflux pumps of the 32 genomes belong to the RND efflux family (**Figure 5**). Most of these genes are shared by all the strains, except for *S. symbiotica* ‘Cinara Cedri’ that only contains 5 of the reported 74 efflux pumps genes. It is not surprising that this strain harbors very few ARGs and efflux pumps because of its reductive genome evolution related to its lifestyle inside the stable and protected niche of the host cell of the aphid *Cinara cedri* (Lamelas et al., 2011; Dutta and Paul, 2012). The highest number of efflux pump genes (57) is present in the Mexican nosocomial strain *S. marcescens* SmUNAM836 sequenced by our group (Sandner-Miranda et al., 2016). Pathogenic strains, including nosocomial strains, show an average number of 50.1 efflux-associated genes, whereas the environmental strains show an average of 44.1 efflux-associated genes and the symbiotic strains only 33.5 efflux-associated genes (**Supplementary Table S2**).

### Acquired Resistance Genes on Plasmids, Transposons, and Integrons

In order to identify genes associated with acquired antibiotic resistance, we screened all the plasmids of the 32 *Serratia* spp. strains. A total of 16 plasmids are reported in the NCBI database for this strain collection (**Supplementary Table S3**). We found 13 plasmid-borne ARGs, 7 β-lactam resistance genes, 3 aminoglycoside resistance genes encoding modifying enzymes, one macrolide resistance gene, one sulfonamide resistance gene, one trimethoprim resistance gene and additionally 3 efflux pump genes associated with resistance to quaternary ammonium compounds, disinfectants and macrolides also on plasmids.

The β-lactam resistance genes class A *bla*_TEM-1,_ *bla*_KPC-2_ and *bla*_SV H-30,_ class B *bla*_IMP-1,_ class C *bla*_ACT-28_ and *bla*_CMY -8,_ and class D *bla*_OXA-9_ were identified in plasmids of nosocomial strains, with the exception of *bla*_ACT-28_, a class C β-lactamase found on plasmid pSF001 of *S. fonticola* GS2, a strain that is associated with a plant. Looking at the nosocomial strains *S. marcescens* SmUNAM836 and *S. marcescens* CAV1429, its plasmids (pSmUNAM836 and pKPC_CAV1492 respectively) harbor class A *bla*_TEM-1_ β-lactamase, which is classified as a broad spectrum β-lactamase (Bush and Jacoby, 2010) and confers resistance to all penicillins and β-lactamase inhibitors. The strain *S. marcescens* CAV1429 also carries two copies of the class A β-lactamase, namely *bla*_KPC-2_, a carbapenemase (Wang et al., 2014), and the *bla*_OXA-9_ gene that encodes an oxacillinase-carbenicillinase (Bojorquez et al., 1998) on plasmid pKPC_CAV1492. The *bla*_SV H-30_ gene, which encodes an ESBL that confers resistance to penicillins and cephalosporines (Bradford, 2001), was located on plasmid pCAV1492-73 of strain *S. marcescens* CAV1492. The imipenem metallo-β-lactamase gene, *bla*_IMP-1_, and the cephalosporin resistance gene, *bla*_*CMY*-8_, were only found on the plasmid pSMC1 of the nosocomial strain *S. marcescens* Sm39.

The aminoglycoside resistance genes *aac(6′)-1b* and *aadA1*, encoding modifying enzymes tobramycin and amikacin 6′ acetyltransferase (Rather et al., 1992) and a streptomycin 3″-adenylyltransferase were found on plasmid pKPC_CAV1492 of the nosocomial strain *S. marcescens* CAV1429. Gene *aadA2*, encoding another streptomycin 3″-adenylyltransferase was found on plasmid pSMC1 of the nosocomial strain *S. marcescens* Sm39. Strain *S. marcescens* Sm39 harbors two copies of the sulfonamide resistance gene *sul1* on plasmid pSMC1.

Three efflux pump genes, *qacE, qacH*, and *msrE*, were found on plasmids of the nosocomial strain *S. marcescens* CAV1492. Two copies of gene *qacE* were found on plasmid pSMC1 of *S. marcescens* SM39. These efflux pumps confer resistance to quaternary ammonium compounds, disinfectants and macrolides, respectively.

As previously reported, it is common to find various ARGs on the same mobile element. Such is the case of the genes *sul1*, *qacE* and some β-lactamases, which are found together in the clinical class 1 integron (Wyrsch et al., 2016). In this study we found two plasmids harboring this integron, plasmid pSMC1 of the nosocomial strain *S. marcescens* Sm39 and plasmid pKPC_CAV1492 of the nosocomial strain *S. marcescens* CAV1492. The class 1 integron is associated with various families of transposons, the Tn*3* transposon present in plasmid pKPC_CAV1492 (Deng et al., 2015) and the Tn*91* transposon present in plasmid pSMC1. Other plasmids harboring heavy metal resistance are also present in some of the *Serratia* spp. genomes (mercury resistance genes in plasmid pCAV1492-73 and pSMC1from the nosocomial strains *S. marcescens* CAV1492 and *S. marcescens* Sm39, respectively, and copper resistance genes in plasmid pCAV1492-199 also from strain *S. marcescens* CAV1492).

The aminoglycoside phosphotransferase gene, *aph(3′)*, was only found on *S. rubidaea* 1122 and *Serratia* sp. FGI94 chromosomes, associated with a transposon. Gene *vanW*, which confers resistance to vancomycin, was found on a transposon of the environmental strain *S. fonticola* DSM4576. These mobile elements have not been previously classified and were inferred in this analysis on the basis of being unique sequences harboring transposases (**Supplementary Table S2**).

Many other genes encoding transposases and integrases were found on all *Serratia* spp. chromosomes, but neither associated with ARGs.

### Efflux Pumps Acquired on the Genomic Islands of *Serratia* spp.

Three efflux pumps were identified on different genomic islands of some of the *S. marcescens* strains using IslandViewer 4 (Bertelli et al., 2017). These genomic islands have not been characterized before. Two of them, MexGHI (Aendekerk et al., 2005) and MexPQ-OprE (Mima et al., 2005), which confer resistance to norfloxacin and macrolides, respectively, showed 82 and 84% homology to the *P. aeruginosa* efflux pumps, indicating a probable horizontal transfer event between these bacteria. The third efflux pump was an allele of *etsABC* that encodes an *E. coli* efflux pump of a putative ABC transport system contained within the pAPEC-O2-ColV plasmid, that confers resistance to macrolides (Johnson et al., 2006). These efflux pumps are also present in other *Serratia* species and most probably on genomic islands as well (**Supplementary Table S2**).

### Resistance Acquired by Gene Mutations

The gene *gyrA* mutation at codon Ser83 was identified in three nosocomial strains, namely *S. marcescens* SmUNAM836, *S. marcescens* Sm39, and *S. marcescens* CAV1492, and also on *S. symbiotica* ‘Cinara Cedri,’ an aphid symbiont. Resistance may arise due to point mutations that result in amino acid substitutions within the topoisomerase genes (*gyrA, gyrB, parC, parE*), with or without a decreased expression of outer membrane porins and overexpression of multidrug efflux pumps (Hopkins et al., 2005). *S. marcescens* SmUNAM836 and *S. marcescens* CAV1492 had an isoleucine residue instead of the expected serine at position 83 of GyrA while the strain *S. marcescens* Sm39 had an arginine residue. *S. symbiotica*, which is a co-obligate symbiont, had a threonine in this position. These mutations are likely to induce a local conformation change of the A subunit of the DNA gyrase, modifying the binding affinity of the quinolones for this enzyme (Yoshida et al., 1990; Weigel et al., 1998). Resistance to one type of quinolone will confer resistance to all (Webber and Piddock, 2001). Neither of the strains had the mutation on gene *folP* that confers resistance to sulfamethoxazole. The *folP* single C→T transition resulting in a Pro→Ser substitution at amino acid position 64 is absent in all genomes (Vedantam et al., 1998). Amino acid substitutions (Asp369Asn and Leu370Ile) in MurA are a major factor in fosfomycin resistance, but neither of the strains showed these mutations (Takahata et al., 2010).

### The *Serratia* spp. Resistome

**FIGURE 6:**
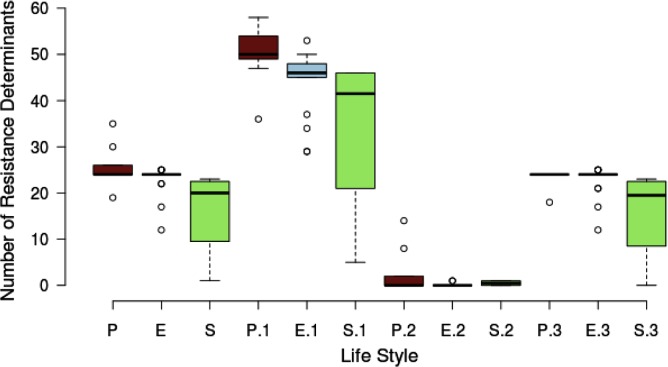
Box plot showing the relationship between the number of resistance determinants and the lifestyle of the strains. There are 3 main lifestyle groups, nosocomial/pathogen [P (brown)], environmental/free living [E (blue)] and environmental/symbiotic [S (green)]. P, E, and S represent the total resistance determinants. P.1, E.1, and S.1 are the total efflux pump genes. P.2, E.2, and S.2 represent the acquired resistance genes and P.3, E.3, and S.3 the intrinsic AGRs.

We classified the resistome according to the niche or lifestyle of the bacteria. We divided the sample of 32 *Serratia* spp. strains into three lifestyles: pathogens (nosocomial and animal pathogens), environmental (bacteria associated with plant, soil, water, or food) and symbiotic bacteria (**Figure 6**). The diagram shows that pathogenic (brown) and environmental strains (blue) do not show a significant different median number of total ARGs, intrinsic and acquired, but symbiotic bacteria (green) do show the smallest number. Regarding efflux pump genes, pathogenic bacteria harbor more of these genes than environmental and symbiotic bacteria. We can appreciate that the difference among resistance gene content between nosocomial/pathogenic and environmental strains is not significant. We believe that this is due to the environment providing a natural reservoir for ARGs.

**FIGURE 7:**
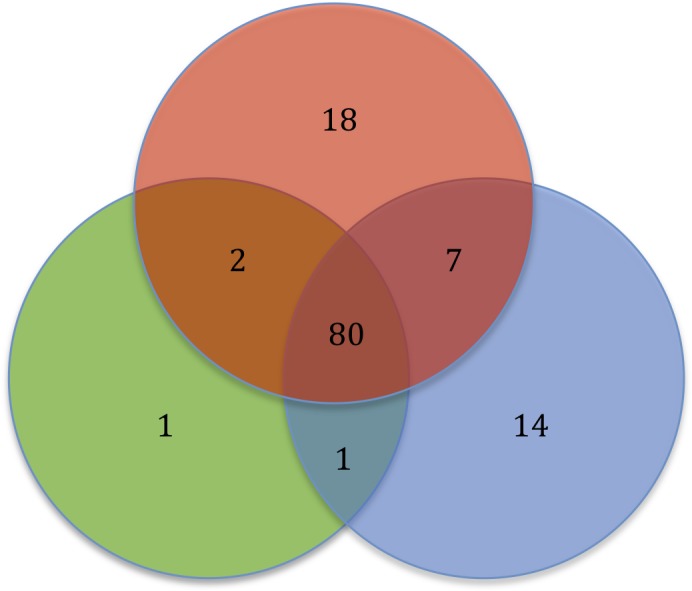
Diagram showing the *Serratia* spp. resistome based on the bacterial niche. Pathogens (red), environmental (blue), and symbiotic (green).

In this comparative genomic study, we report that the *Serratia* spp. resistome consists of genes shared by the majority of the strains and genes exclusive of niche. With the analysis of 32 *Serratia* spp. genomes, we report a resistome for the *Serratia* genus totaling 123 ARGs, 49 intrinsic and acquired ARGs, and 74 efflux pump-associated genes. Of the 123 ARGs, 80 are present in one or more strains from each niche, 7 are shared by pathogens and environmental strains only, 2 are shared by pathogens and symbiotic strains, and one is shared by environmental and symbiotic strains. 18 ARGs were found exclusively in pathogenic strains, 14 in environmental strains and one in symbiotic strains (**Figure 7**).

The polymyxin resistance operon and the genes *catA*, *bacA, fosA* and *hslJ*, which confer resistance to chloramphenicol, bacitracin, fosfomycin and novobiocin, respectively, are among the shared ARGs. In addition, the aminoglycoside acelyltransferase gene, *aac(6′)-Ic*, the aminoglycoside phosphotransferase gene, *aph(3″)*, and the quinolone resistance gene, *qnr*, are shared by the majority of the strains, regardless of their lifestyles. The class C β-lactamase gene, *ampC*, and all the regulatory genes were also found in most of the strain chromosomes. Most of the strains share most of the genes associated with efflux systems.

The 18 ARGs exclusive of pathogens are genes located on plasmids, mainly β-lactamase coding genes, with the exception of the alleles of the efflux pumps EmrAB and AcrD, which are located on the chromosome (**Supplementary Table S2**). The 14 ARGs exclusive of environmental strains are genes found on the chromosome, all of intrinsic resistance except for the vancomycin resistance gene *vanW* found on a transposon. Only one ARG was found to be exclusive of a symbiotic strain, an allele of the efflux pump AcrF that confers resistance to ciprofloxacin. Five efflux pumps genes, *mexP, mexQ, opmE* and *triC*, from the RND family conferring resistance to macrolides, fluoroquinolones and triclosan, respectively, and the gene *mdsC*, which is part of the efflux pump MdsAB (absent in this sample), are shared by pathogen and environmental strains. The aminoglycoside phosphotransferase gene *aph(3′)* and the mutation of *gyrA*, which confers resistance to quinolones, were located in pathogens and symbiotic strains. Only the streptogramin efflux pump gene, *vgaC*, is shared by environmental and symbiotic strains and is absent in pathogens.

### Similarities and Dissimilarities Between the *Serratia* spp. Resistomes

**FIGURE 8:**
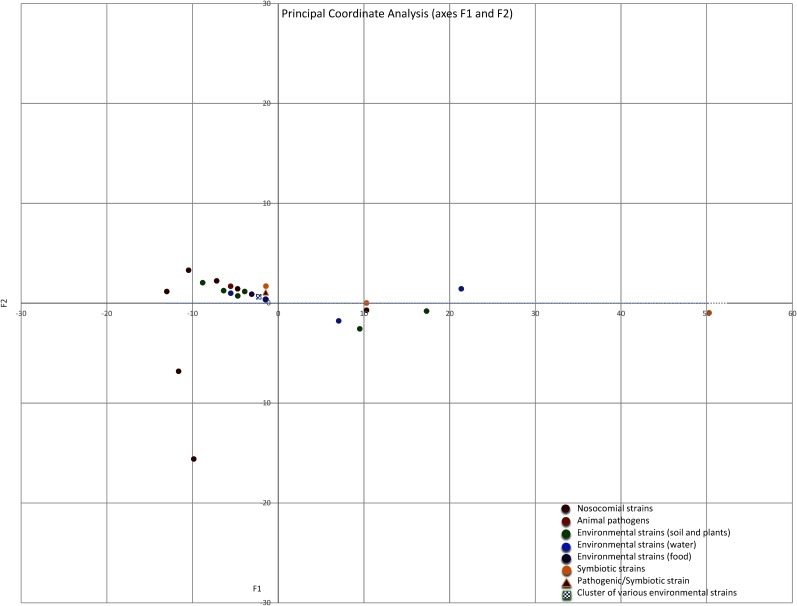
Principal Coordinate Analysis (PCoA) plot depicting Euclidean distances between resistomes of nosocomial strains (red), animal pathogens (orange), environmental associated with soil and plants (green), environmental isolated from water (blue), environmental isolated from food (purple), and environmental symbiotic bacteria (yellow) calculated using ARGs counts. The blue and green square represents a group of many strains isolated from water and soil/plants, the triangle represents de pathogen/symbiotic strain *S. nematodiphila.*

The intrinsic and acquired ARGs, and the efflux pump-associated genes, which were identified in the 10 *Serratia* species and in the 4 non-characterized strains, were grouped according to the lifestyle of each strain in a matrix of similarity/dissimilarity. A PCoA analysis was performed to visualize the similarities of the *Serratia* spp. resistomes according to the 3 previously mentioned ecological niches. The environmental strains were sub-divided into strains associated with soil and plants, strains isolated from water and strains isolated from food. The plot of Euclidean distances shows that the resistomes of nosocomial and pathogenic strains are similar to those isolated from the natural environment based on the number of ARGs (**Figure 8**). The most dissimilar resistome was from the co-obligate aphid symbiont, followed by the resistome of strain *Serratia* sp. ATCC 39006 isolated from salt marsh water and a questionable member of the genus *Serratia*, the resistome of the fungus garden symbiont *Serratia* sp. FGI94 (reclassified in this work as *S. rubidea*) and the resistome of *C. multitudinisentens* RB-25 isolated from soil. Nosocomial strains with the highest number of acquired ARGs, *S. marcescens* Sm39 and *S. marcescens* CAV1429, also showed dissimilarity with respect to the others. It is not surprising that nosocomial strains present the highest number of acquired ARGs as they are subject to a strong selection pressure due to frequent antibiotic use in hospitals.

It would be interesting to include a new set of *Serratia* isolates to be collected from animal and environment sources that could confirm the selection pressure of the indiscriminate use of antibiotics in the hospital environment on nosocomial strains and address unsolved issues on the zoonotic origin of antimicrobial resistance genes (Cloeckaert et al., 2017).

## Discussion

The microbial pan-genome is the cumulative number of different genes found within genomes of a particular taxonomic rank, usually within a species, though this can be extended to higher levels, such as a genus (Tettelin et al., 2008). It contains the core genes, common to all strains of the study, the accessory genome containing genes present between two and *n*–1 strains, and the unique or singleton genes present only in a single strain. Inside the pan-genome, we can study different features, such as the resistome (Rouli et al., 2015). The pan-genome size, and whether it is open or closed, depends in part on bacterial lifestyle. Large and open pan-genomes are associated with bacterial species that live within communities and that are prone to horizontal gene exchange. However, the more genomes used to predict the pan-genome, the larger its predicted size, due to the contribution of rare genes (Tettelin et al., 2008). Here, we report an observed pan-genome of 12,347 clusters of genes for a collection of 32 strains of the genus *Serratia*, which we consider a relatively large pan-genome based on the small sample size of this genus. It is also considered to be relatively large when compared to the pan-genome of 5,398 clusters for 50 *Streptococcus* genomes from 14 species (Contreras-Moreira and Vinuesa, 2013), and around 10,000 family genes for the *Salmonella* genus (Jacobsen et al., 2011). On the other hand, for the genus *Vibrio*, there is a report of a pan-genome consisting of 26,504 genes for 43 different species (Thompson et al., 2009), which is a very large pan-genome and reflects the high diversity of this genus. For the small genome of genus *Mycoplasma* there is an estimate of a pan-genome consisting of 8,000 genes, which is very large if we consider the small genome size (0.5 and 1.4 Mb) of this genus; this huge pan-genome size may be the reflection of their diverse lifestyles (Liu et al., 2012). Large pan-genomes indicate that these genomes have a considerable amount of unique sequences, such as mobile elements, genomic islands, transposons or prophages.

The phylogenetic analysis based on the pan-genome of the 32 *Serratia* spp. is perfectly supported by our maximum likelihood core-genome phylogeny. All *S. marcescens* strains group together with the uncharacterized strains *Serratia* sp. YD25, *Serratia* sp. FS14 and *Serratia* sp. SCB1, suggesting that they should be reclassified as members of the *S. marcescens* species. This is clearly supported by our cgANIb estimates which are >98% in all cases. It is worth noting that *S. nematodiphila* DSM 21420 clusters in the same subgroup as *S. marcescens* Db11 and *Serratia* sp. SCB1, which are both insect pathogens. This fact can be explained due to the dual quality of *S. nematodiphila*, which is a nematode symbiont, but a pathogen of the insect that is parasitized by the nematode (Kwak et al., 2015). The evidence presented in this study from the core-genome phylogeny and cgANIb analyses strongly suggest that strains DSM 21420 and SCB1 should be reclassified as *S. marcescens. S. phymuthica*, *S. liquefaciens* and *S. proteamaculans* strains are placed in the sister group to *S. marcescens* strains. Two of the *S. liquefaciens* strains are nosocomial pathogens. This grouping is congruent with previous studies (Abebe-Akele et al., 2015; Li et al., 2015). *Serratia* sp. FGI94, which is a fungus symbiont strain and *S. rubidaea* 1122 isolated from a patient in China are placed together in group C. The evidence from the core-genome phylogeny and cgANIb data clearly indicate that FGI94 belongs to the *S. rubidaea* species and that it is wrongly classified. Both have similar chromosome size and harbor the aminoglycoside phosphotransferase gene *aph(3′)* on a transposon. The most distantly related strains are *C. multitudinisentens* [recently reclassified as *C. multitudinisentens* (Ee et al., 2016)] isolated from the soil of an ex-landfill site, *Serratia* sp. ATCC 39006 isolated from water and the co-obligate aphid symbiont *S. symbiotica* ‘Cinara Cedri’. The genomes of these 3 strains harbor the fewest ARGs on their chromosomes. Based on their <85% cgANIb values when compared to the other *Serratia* genomes, their classification in this genus should be carefully revised.

Bacterial antimicrobial resistance occurs by one or a combination of different mechanisms: a reduction in antibiotic passage through the bacterial outer membrane preventing access to the target; modification of antibiotic targets by modifying enzymes; antibiotic hydrolysis; and increased transport of the antibiotic out of the cell by efflux pumps (Shaikh et al., 2015; Munita and Arias, 2016). The modulation of resistance to certain antibiotics depends also on the activation of regulatory genes, mutations of specific genes, intrinsic differences in the structure of the outer membrane, such as porin alterations that reduce the entry of antimicrobials, such as carbapenems (Gupta et al., 2011), or due to the acquisition of novel resistance genes by HGT (Blair et al., 2014). It is important to recognize that the concept of antimicrobial resistance is a phenomenon with many layers of complexity. Due to this complexity, a resistance genotype does not necessarily produce a resistant phenotype all the time; an example of this is illustrated with the *aac (6′)-Ic* gene. The *aac(6′)-Ic* gene was cloned from *S. marcescens* (Shaw et al., 1992) and DNA hybridization analysis demonstrated that all *S. marcescens* strains carried the *aac(6′)-Ic* gene, however, not all presented the AAC(6′)-Ic resistance profile (Shaw et al., 1993). In this comparative study, we looked for all classes of genes involved in the different antibiotic resistance mechanisms mentioned above, including regulatory genes (detailed information is shown in **Supplementary Table S2**).

The resistome of *Serratia* spp. is composed mainly by intrinsic resistance genes (structural), mostly by genes encoding efflux pumps systems. These pumps, besides conferring resistance to antibiotics, have other important physiological roles and therefore, have greater clinical relevance than is usually attributed to them (Piddock, 2006). These groups of bacteria harbor a “natural” resistance to most of the antibiotics, such as the β-lactam family, due to the presence of the β-lactamase AmpC (Jacoby, 2009), to polypeptide antibiotics through the operon *arn*, and to quinolones by having the gene *qnr*. This resistance to a wide spectrum of antimicrobials makes it difficult to select an appropriate treatment for *Serratia* spp. infections. Surprisingly, the *Serratia* genus lacks resistance genes for trimethoprim and sulfonamides (with the exception of *S. marcescens* CAV1492 which has acquired the trimethoprim and sulfonamide resistance genes *dfr5* and *sul1* on one of its plasmids, and *S. marcescens* Sm39 which harbors the sulfonamide gene *sul1* on plasmid pSMC1). *Serratia* genomes also lack the *folP* mutation in position 64 that results in a Pro→Ser aminoacid substitution and that confers sulfonamide resistance (Vedantam et al., 1998), which makes this combination of antibiotics a good choice in the treatment of bacterial infections caused by this genus. One evolutionary hypothesis for the lack of these resistance genes in this genus could be based on the antibiotic resistance cost. Resistance is often associated with reduced bacterial fitness and the reduction in antibiotic use will benefit the fitter susceptible bacteria (Andersson and Hughes, 2010). The combination of these antimicrobials has been suspended in many countries relaxing the selection pressure on these genes and leading to gene loss. An alternative hypothesis could be that this genus is intrinsically susceptible to trimethoprim-sulfamethoxazole and that they have never carried these genes on their chromosomes. The presence of these ARGs on the plasmids of two nosocomial *S. marcescens* strains can be explained by HGT events and by genetic co-selection, where the use of one antibiotic will exert selection pressure on all the ARGs that are on the same mobile element.

Our results show that most of the resistomes of pathogenic and environmental bacteria of the genus *Serratia* are very similar in the number of ARGs shared, being the content of horizontally transferred genes what determine the differences with the nosocomial strains. To date there are around 350 *S. marcescens* genomes in the NCBI database, and more than 450 for the whole genus, but only 10.8% of these genomes are assembled and annotated completely. We decided to work with complete genomes retrieved from NCBI’s GenBank and RefSeq repositories to avoid biases introduced by highly fragmented genomes in phylogenomic inference and comparative pan-genomic. This can lead to a potential limitation of our results; nevertheless, we presume that the results obtained here are a suitable approximation for the resistome of this bacterial genus.

The nosocomial strains with the highest number of acquired ARGs are *S. marcescens* CAV1429 and Sm39 with 14 and 8 genes on their plasmids, respectively, mostly β-lactamases. We also found that both pathogenic and environmental strains share genes with high nucleotide identities confirming what has been previously observed, in that the environmental isolates from soil (D’Costa et al., 2006), plants and water represent a natural reservoir for ARGs (Riesenfeld et al., 2004). While ARGs of environmental isolates may originally have had different functions aside from conferring resistance to antibiotics produced by other competing bacteria, these genes have now been acquired as resistance genes in pathogenic bacteria via HGT (Berglund, 2015). There are several other known factors that promote resistance in susceptible bacteria: selection pressure placed on susceptible microbes through the use of therapeutic agents, over-prescription, self-medication, treatment non-compliance, use of antibiotics in food-producing animals and in agriculture in general (Knobler, 2003), and an increase in antimicrobial residues found in the environment, most particularly in water (Harris et al., 2013). It is important to raise awareness concerning the excessive use of antibiotics in agricultural, poultry and livestock industries by creating specialized surveillance institutions that coordinate the health, food and environmental sectors, enabling the identification of the many routes for both dissemination and acquisition of ARGs from agricultural and environmental microbial communities to human pathogens, preventing nosocomial outbreaks. It is also crucial to identify the intrinsic resistance profile of a given microorganism or species in order to select the most suitable antimicrobial treatment not forgetting to link the laboratory-based phenotype antibiogram to the genomic data to understand which ARGs are in circulation and which represent a threat to the medical community (McArthur and Wright, 2015). Due to the fact that the *Serratia* species have a high number of efflux systems, no matter how many times a new antibiotic molecule is generated, resistance will persist. Therefore, new strategies for generating effective treatments have to take into account other targets, for example, inhibiting the expression of regulatory genes of the efflux pumps.

Whole genome sequencing and metagenomics are opening the door to rapid genotype-based resistance diagnosis (Bertelli and Greub, 2013; Wyrsch et al., 2016) that in turn are helping in the rapid detection and effective treatment of bacterial infections.

## Conclusion

Awareness of the increasing problem related to antibiotic resistance has been a great concern in public health in recent years. Combating this problem requires an understanding of the mechanisms, evolution and spread of ARGs and the manufacture of new drugs that can circumvent resistance. Members of the *Serratia* genus are gram-negative bacteria of the *Enterobacteriacea* family that have been isolated from various ecological niches, such as soil, water and hospitals. *S. marcescens* has emerged recently as an opportunistic pathogen associated mostly with nosocomial infections. *Serratia* spp. infections pose a major health problem due to their high multidrug resistance, mainly because of the high number of efflux pump genes present in their genomes. This makes it difficult to choose suitable treatments. Knowing the genomic composition of *Serratia* spp. and the genes that confer resistance, will allow timely treatments to be determined and avoid those antibiotics to which the bacterium has a natural or acquired resistance.

## Author Contributions

LS-M performed the experiments, did the phylogenetic analysis, analyzed the data, and wrote the paper. PV performed the bioinformatic analysis. AC the final approval of the version of the manuscript to be published. RM-E conceived and designed the experiments, contributed in writing the paper, and gave the final approval for the publication.

## Conflict of Interest Statement

The authors declare that the research was conducted in the absence of any commercial or financial relationships that could be construed as a potential conflict of interest.

## Footnotes

1 https://github.com/vinuesa/get_phylomarkers

## Supplementary Material

FIGURE S1 Venn diagram of the consensus core-genome of the 32 *Serratia* spp. genomes generated by COG, OMCL, and BDBH algorithms.

FIGURE S2 Pairwise core-genome average nucleotide identity values from OMCL clusters (cgANIb-OMCL). The colors in the heat map represent pairwise ANI values, with a gradient from light yellow (high identity) to red (low identity).

FIGURE S3 Venn analysis of the pan-genome of the 32 *Serratia* spp. genomes generated by COG and OMCL algorithms.

TABLE S1 List of the 32 *Serratia* spp. genomes used in this study, source of isolation, geographic region, continent, NCBI genome reference number, and genome size.

TABLE S2 Total number of ARGs and efflux pumps identified in the 32 *Serratia* genomes. Genes found on the chromosome in white color, genes found on plasmids in orange color, genes found on transposons in blue color, genes found on probable genomic islands in purple color, regulatory genes in pink color and genes with mutations that confer resistance in green color.

TABLE S3 Plasmids and acquired ARGs of the 32 *Serratia* spp. genomes. β-lactam resistance genes: blue, aminoglycoside resistance genes: red, macrolide resistance gene: yellow, sulfamide/trimethoprim resistance gene: pink, quaternary ammonium compounds: green.
