# Supplementary figures and images for: The Genomic Basis of Intrinsic and Acquired Antibiotic Resistance in the Genus Serratia
Source: Front Microbiol. 2018 May 11;9:828. doi: 10.3389/fmicb.2018.00828 (PMC5958200; doi:10.3389/fmicb.2018.00828)

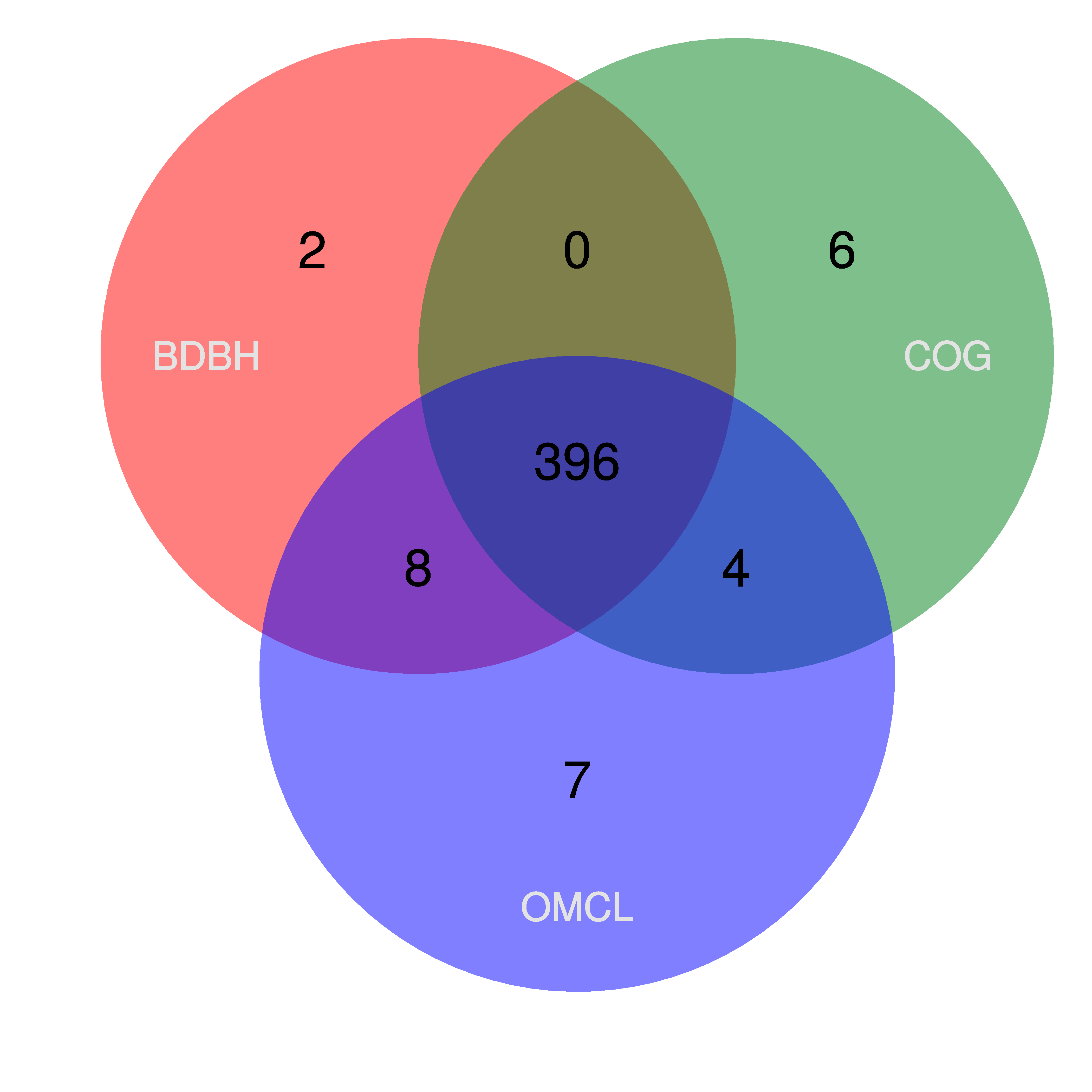

Supplement: FIGURE S1 — Venn diagram of the consensus core-genome of the 32 Serratia spp. genomes generated by COG, OMCL, and BDBH algorithms. [file Image_1.tif]

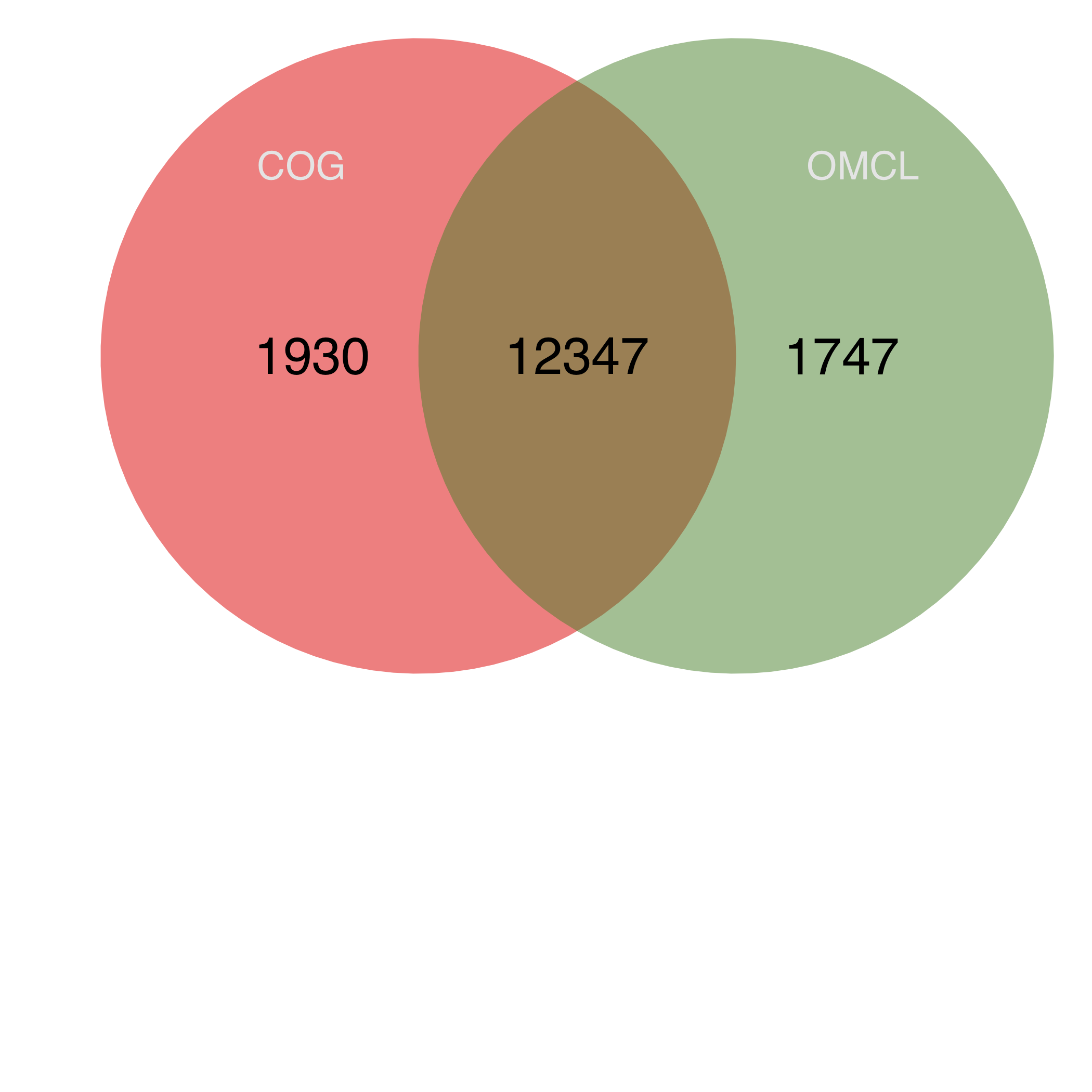

Supplement: FIGURE S3 — Venn analysis of the pan-genome of the 32 Serratia spp. genomes generated by COG and OMCL algorithms. [file Image_3.tiff]
